# The ovine *HIAT1* gene: mRNA expression, InDel mutations, and growth trait associations

**DOI:** 10.3389/fvets.2023.1134903

**Published:** 2023-04-17

**Authors:** Yunyun Luo, Zhanerke Akhatayeva, Cui Mao, Fugui Jiang, Zhengang Guo, Hongwei Xu, Xianyong Lan

**Affiliations:** ^1^Key Laboratory of Animal Genetics, Breeding and Reproduction of Shaanxi Province, College Animal Science and Technology, Northwest A&F University, Yangling, Shaanxi, China; ^2^Shandong Key Lab of Animal Disease Control and Breeding, Institute of Animal Science and Veterinary Medicine, Shandong Academy of Agricultural Sciences, Jinan, China; ^3^Bijie Animal Husbandry and Veterinary Science Research Institute, Bijie, China; ^4^College of Life Science and Engineering, Northwest Minzu University, Lanzhou, China

**Keywords:** sheep, HIAT1 gene, insertion/deletion (InDel), growth traits, marker-assisted selection (MAS)

## Abstract

**Background:**

The *hippocampal abundant transcript 1 (HIAT1)* gene, also known as *major facilitator superfamily domain-containing 14A (MFSD14A)*, encodes for a transmembrane transporter protein and has been previously shown to be associated with milk production in buffalo and sheep breeds, as well as growth traits in chicken and goats. However, tissue level distribution of the ovine HIAT1 gene, as well as its effect on body morphometric traits in sheep, has yet to be studied.

**Methods:**

The *HIAT1* mRNA expression profile of Lanzhou fat-tailed (LFT) sheep was determined by quantitative real-time PCR (qPCR). A total of 1498 sheep of three indigenous Chinese sheep breeds were PCR-genotyped for polymorphisms of *HIAT1* gene. Student's t-test was used to observe the association between the genotype and sheep morphometric traits.

**Results:**

*HIAT1* was widely expressed in all examined tissues, and was particularly abundant in the testis of male LFT sheep. Additionally, a 9-bp insertion mutation (rs1089950828) located within the 5'-upstream region of *HIAT1* was investigated in Luxi black-headed (LXBH) sheep and Guiqian semi-fine wool (GSFW) sheep. The wildtype allele frequency 'D' was found to be more prevalent than that of the mutant allele ‘I'. Furthermore, low genetic diversity was confirmed in all sampled sheep populations. Subsequent association analyses indicated an association between the 9-bp InDel mutation of interest and the morphometric traits of LXBH and GSFW sheep. Furthermore, yearling ewes with a heterozygous genotype (ID) demonstrated smaller body sizes, while yearling rams and adult ewes with the heterozygous genotype were found to have overall better growth performance.

**Conclusion:**

These findings imply that functional InDel polymorphism (rs1089950828) has the potential to be utilized for marker-assisted selection (MAS) of growth traits in domestic Chinese sheep populations.

## 1. Introduction

The final grown performance of sheep and other domesticated animals is affected by multiple factors including environmental, nutritional, and genetic influences (1). Using high-throughput whole genome sequencing, RNA sequencing, and molecular biology techniques, a number of candidate functional genes and genetic variants linked to important growth traits in livestock were found and used for the purposes of molecular-assisted and omics-assisted breeding (2–6). Among the numerous genome variants including SNPs (single nucleotide polymorphisms), CNVs (copy number variations), and other genetic markers, numerous studies have demonstrated that InDels (insertion/deletion) function by regulating gene translation or altering a protein and are related to differences in the characteristics of individual animals (7–9).

The *hippocampal abundant transcript 1* (*HIAT1*) gene is also known as the *major facilitator superfamily domain 14A* (*MFSD14A*) gene (10). The HIAT1 protein contains a conserved sugar transporter motif as well as 12 transmembrane alpha helices (TMs) linked by hydrophilic loops, and belongs to the major facilitator superfamily (MFS) of secondary protein transporters (11). The MFS protein superfamily is the largest secondary transporter family present in a broad variety of species from archaea to mammals and plays an essential role in the exchange of cellular materials and energy metabolism, which facilitates the transport of many substrates across both cytoplasmic and internal cell membranes (12). The expression of both *MFSD1* and *MFSD3* genes has previously been shown to be significantly different in both the cerebellum and brain of mice after they were subjected to starvation and high-fat diets. This suggests that these genes are regulated at the nutrient level and may be related to nutrient intake and transport (13). Two members of the MFS gene family, *MFSD6* and *MFSD10* were found to be related to the energy metabolism of cells. Expression levels of these two genes were found to be down-regulated when the cellular energy consumption rate increased or when cellular energy was significantly depleted (14). These findings suggest that *HIAT1* may also be related to fatty acid uptake and transport.

Chinese scholars have found that *HIAT1* affects buffalo milk fat synthesis mainly through regulation of cellular membrane transport functions (15). Moreover, studies have shown that the HIAT1 protein has the capacity to transport solute from the blood stream that is required for spermiogenesis, thus disruption of this gene has been shown to cause round-headed sperm and sterility in male mice (16). *HIAT1* has previously been shown to be commonly expressed in multiple tissues types and highly expressed in testis, suggesting that it has extensive biological functions (17). *HIAT1* is expressed in brown adipose tissue and is negatively correlated with the process in which glucose is converted to fat (18). Additionally, polymorphisms in *HIAT1* have been shown to affect milk traits in buffalo as well as growth in goats (15, 19). However, to date, there are few studies that have reported on the function of *HIAT1* in indigenous Chinese sheep populations.

In order to further improve the production features of native Chinese sheep populations, the current study aimed to detect *HIAT1* expression features in Lanzhou fat-tailed (LFT) sheep, explore InDel variations of the *HIAT1* gene in three indigenous Chinese sheep breeds, which included Luxi black-headed (LXBH) sheep from east China, Guiqian semi-fine wool (GSFW) sheep from southwest China, and LFT sheep from northwest China, as well as conduct association analyses between sheep genotypes and growth traits to provide a basis for use of the *HIAT1* gene for sheep selection and breeding.

## 2. Materials and methods

### 2.1. Animals and ethics statement

Twelve tissue samples were collected from three 6-month-old male Lanzhou fat-tailed sheep and include heart, liver, spleen, lung, kidney, testis, longissimus dorsi muscle, tail adipose, perirenal fat, subcutaneous fat, small intestine, and rennet stomach (Yongjing county, Gansu Province). A total of 1498 indigenous Chinese sheep were used for DNA experiments and included Luxi black-headed sheep (*n* = 616) raised in Liaocheng city, Shandong province; Guiqian semi-fine wool sheep (*n* = 824) raised in Bijie city, Guizhou province; and Lanzhou fat-tailed sheep (*n* = 58) provided by Gansu Ruilin Science and Technology Breeding Company (Yongjing county, Gansu province). All tissue samples were frozen in liquid nitrogen and stored at −80°C for RNA and DNA isolation. Relevant body morphometric traits were recorded, including body weight, chest circumference, height at hip cross, body height, body length, cannon circumference, hip width, abdominal circumference, chest depth, and chest width. Animal experiments were conducted in line with the guidelines for the care and use of animals and approved by the Animal Ethics Committee of Northwest A&F University.

### 2.2. DNA isolation, primer design, and InDel genotyping

Genomic DNA was isolated following previously established protocols (20, 21). Total RNA and genomic DNA concentrations were determined using Nanodrop 2000. DNA was diluted to a concentration of 20 ng/μL and stored at −40°C. Thirty DNA samples were randomly selected from each breed to construct three genomic DNA pools for investigation of *HIAT1* variations.

Three upstream gene variants were screened from the Ensembl database, and then several primers (Table 1) were designed using Primer Premier 5 software and synthesized by Sangon Biotech (Xi'an, China) based on reference sequences in ovine *HIAT1* (NC 056054.1). Polymorphic fragments were amplified using the polymerase chain reaction (PCR) method and utilized a touch-down program and reaction volumes as described in previous studies (8, 22). PCR products were then separated by electrophoresis on a 3.5% agarose gel stained with GoldView. The genotype of each individual sheep was analyzed.

**Table 1:** Primers used for InDel detection and qPCR analysis of ovine *HIAT1*.

**Names**	**ID**	**Alleles**	**Primer sequences (5' to 3')**	**Sizes (bp)**
P1-ins-5bp	rs598326621	-/TTAAG	F: TTCCTTCACTCCTTAAGACTTCG R: TTTGATTGTGATGACTGTACTGT	89/84
P2-del-5bp	rs604922868	TCAGT/-	F: CGCAATTCCCCCATTAAAT R: GCAAAGAGTCGGACACGAC	109/104
P3-ins-9bp	rs1089950828	-/GTCCAGTGG	F: TTCCCTGTTCATCACCAACTC R: ACCTTTTCTTTATTCCCTGCC	148/139
*HIAT1*	XM_004002229.5	-	F: TACTGCTGGCTCTGCTTGTTGC R: TACTGTGAGGATGGCTGTGACTACC	108
*GAPDH*	NM_001190390.1	-	F: CCTGCCAAGTATGATGAGAT R: TGAGTGTCGCTGTTGAAGT	119

### 2.3. RNA isolation, cDNA synthesis, and quantitative real-time PCR

Total RNA was extracted from tissue samples using TRIzol reagent, and cDNA was synthesized according to manufactures' instructions using an Evo M-MLV RT Kit with gDNA Clean for qPCR II (Accurate biology Co., Ltd, Changsha, China). A LightCycler 96 real-time PCR system (Roche, Switzerland) was used to run the qPCR program. qPCR reactions (10 μL) contained 5 μL 2 × ChamQ SYBR qPCR Master Mix (Vazyme, Nanjing, China), 0.2 μL of each primer, and 4.6 μL cDNA (1/100 dilution). qPCR conditions were as follows: 95°C for 180 s, followed by 40 amplification cycles lasting 40 s each (95°C for 10 s and 60°C for 30 s). Gene expression was quantitated using the 2^−ΔΔ*Ct*^ method.

### 2.4. Bioinformatics and statistical analysis

Differing amino acid sequences alignment were made using DNAMAN 6.0 software. The percent similarity of amino acid sequences was analyzed by NCBI-Blast online software. Using AliBaba2.1 (http://gene-regulation.com/pub/programs/alibaba2/), predictions of transcription factor binding to the mutant ovine *HIAT1* gene sequence were generated (23). Population genetic indices, as well as allelic and genotypic frequencies, were determined. χ^2^ test was performed to determine whether the gene variants are within Hardy-Weinberg equilibrium (HWE) and analyze the difference in genotype distributions amongst each sheep population. The statistical model used was Y_*ij*_ = μ + G_*i*_ + ε_*ij*_, where Y_*ij*_ = observed growth traits, μ = population mean, G_*i*_ = fixed effect of the genotype, ε_*ij*_ = random error. If there were only two genotypes, student's *t*-test was performed to determine the effect of different genotypes on economic traits.

## 3. Results

### 3.1. Multiple sequence alignment and mRNA expression

**Figure 1:**
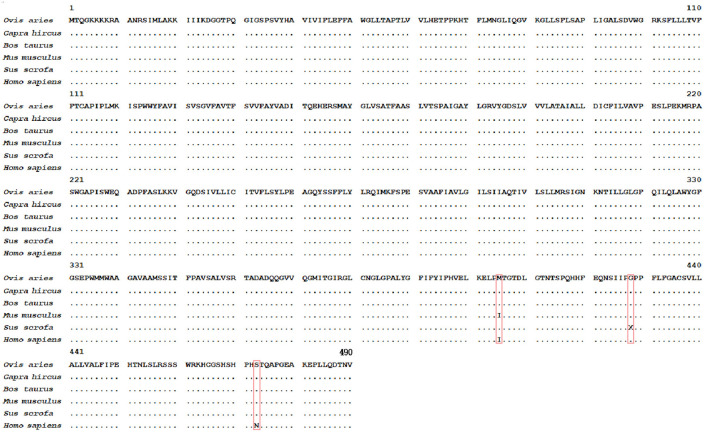
Amino acid sequence alignment of *HIAT1* in different mammalian species. The red boxes denote the parts of the sequence that are unable to be matched with the *Ovis aries* sequence. Note: **•** denotes the same amino acid.

**Figure 2:**
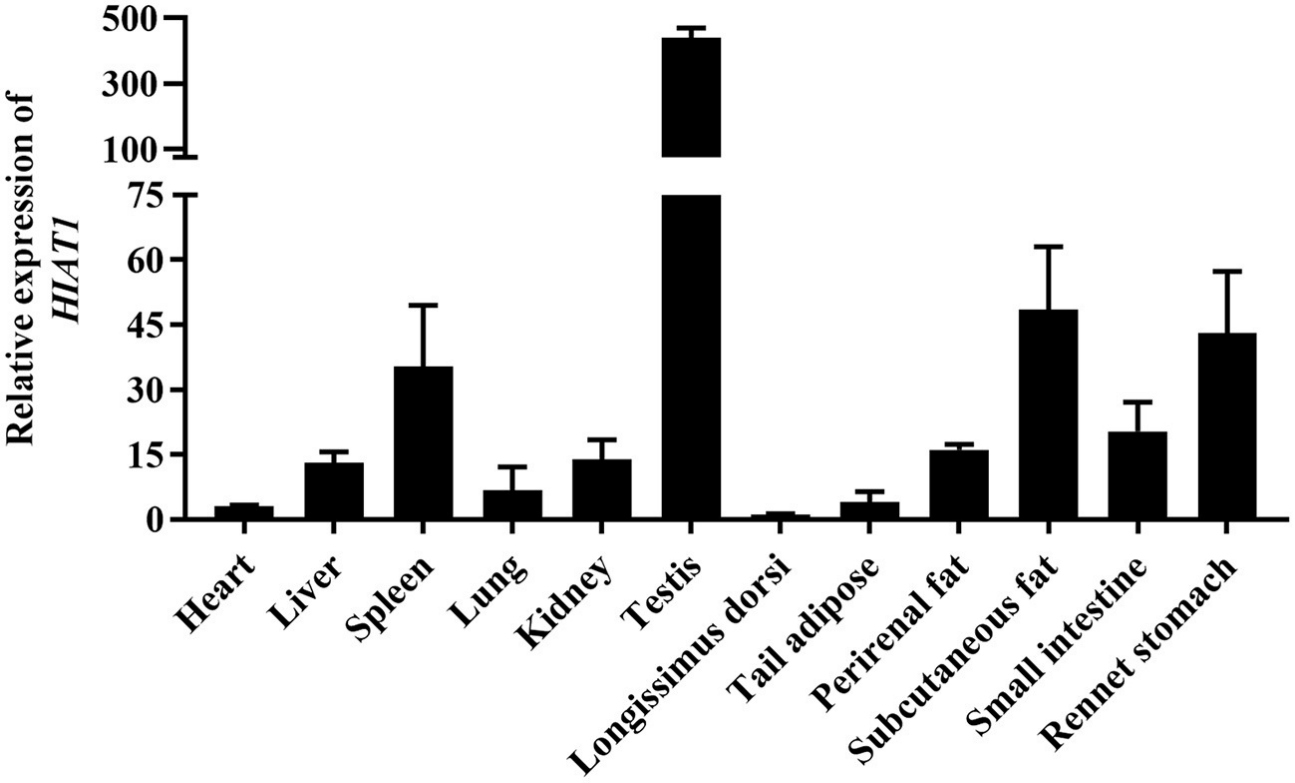
*HIAT1* mRNA expression patterns detected by qPCR in Lanzhou fat-tailed sheep.

The results of NCBI-BLASTP sequence analysis showed that the *HIAT1* in *Ovis aries* (Genbank: XP_004002278.1) shares 100%, 100%, 99.80%, 99.80% and 99.59% similarity with that of *Capra hircus* (XP_017901305.1), *Bos taurus* (NP_001095508.1), *Mus musculus* (NP_032272.2), *Sus scrofa* (XP_013852805.2) and *Homo sapiens* (NP_149044.2), respectively (Figure 1). Additionally, qPCR results revealed that *HIAT1* was expressed in most sheep tissues, especially in the testis of male LFT sheep (Figure 2), which coincided with expression and distribution in human tissues.

### 3.2. Detection and genetic parameter analysis of InDel polymorphisms within the *HIAT1* gene

**Figure 3:**
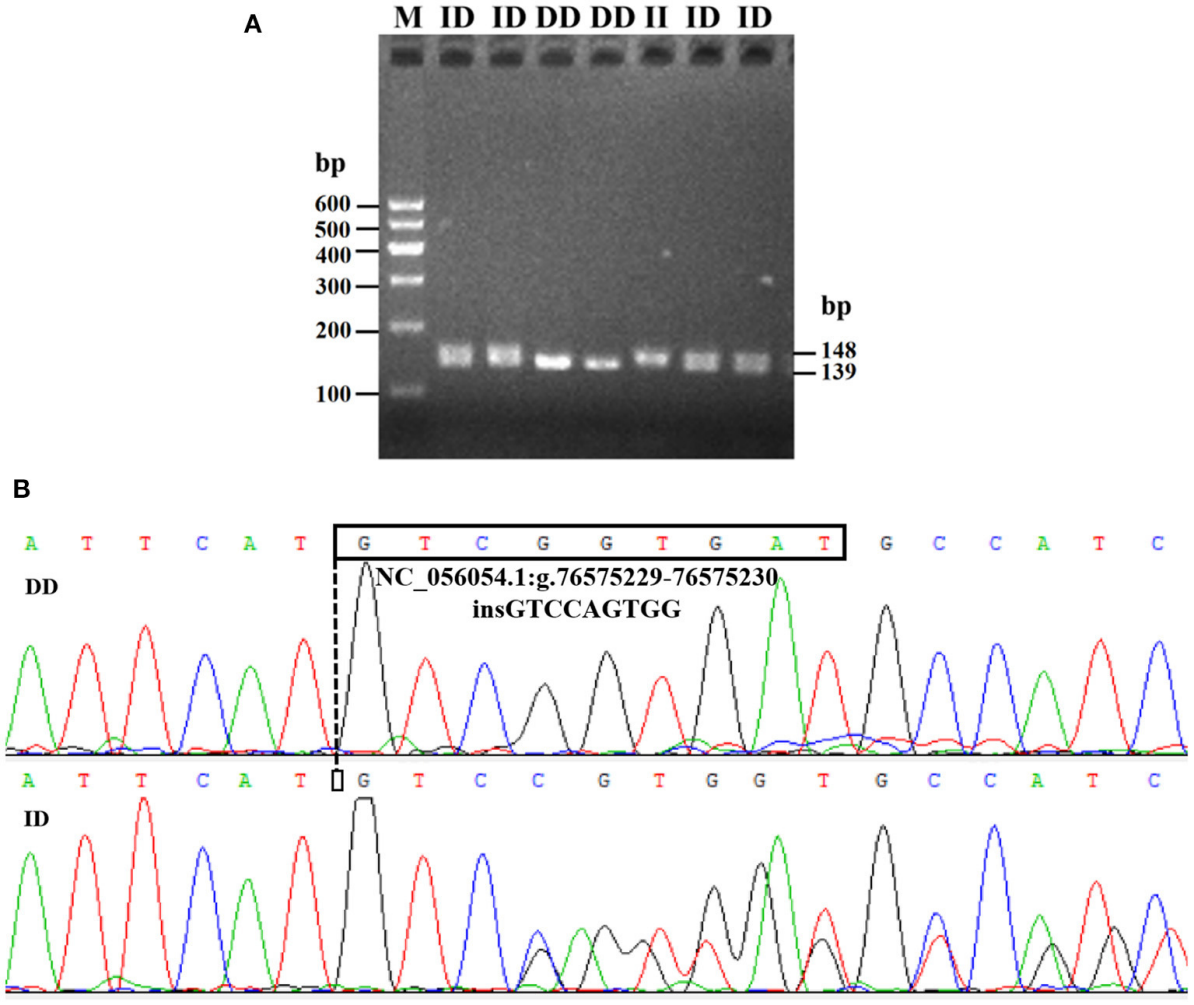
Electrophoresis gel **(A)** and sequence chromatograms **(B)** of the P3-ins-9bp InDel within the ovine *HIAT1* gene.

Electrophoresis patterning and sequence mapping of the P3-ins-9bp InDel showed polymorphisms in the 5'-upstream region of *HIAT1* for both Luxi black-headed sheep and Guiqian semi-fine wool sheep. There were no detected *HIAT1* polymorphisms found in Lanzhou fat-tailed sheep (Figure 3). Genotyping analyses showed that the *HIAT1* P3-ins-9bp locus contained three genotypes (the homozygous insertion type, II, 148 bp; the homozygous deletion type, DD, 139 bp; and the heterozygous mutation type, ID, 148 bp and 139 bp) for both Luxi black-headed sheep and Guiqian semi-fine wool sheep; however, only one genotype (DD) was discovered in the Lanzhou fat-tailed breed.

The genotypic frequency, allelic frequency, and population genetic indices were subsequently calculated and shown in Table 2. The DD genotype frequency was found to be much higher than both ID and II genotypes. Amongst all sheep breeds tested, the P3-ins-9bp locus was found to have low genetic polymorphism (*PIC* < 0.25) while maintaining Hardy-Weinberg equilibrium (*P* > 0.05). χ^2^ testing revealed that there were significant differences both in the genotypic and allelic frequency distributions of the P3-ins-9bp locus amongst all three populations (*P* < 0.05) (Table 3).

**Table 2:** Genotypic frequencies, allelic frequencies, and genetic diversity of the P3-ins-9bp InDel.

**Breeds (n)**	**Genotypic frequencies**			**Allelic frequencies**		**HWE *P-*value**	**Population parameters**		
	**II (n)**	**ID (n)**	**DD (n)**	**I**	**D**		**He**	**Ne**	**PIC**
LXBH (616)	0.006 (4)	0.156 (96)	0.838 (516)	0.084	0.916	*P*>0.05	0.155	1.183	0.143
GSFW (824)	0.004 (3)	0.080 (66)	0.916 (755)	0.044	0.956	*P*>0.05	0.084	1.091	0.080
LFT (58)	0	0	1.000 (58)	0	1.000	*-*	0	1.000	0

LXBH, Luxi black-headed sheep; GSFW, Guiqian semi-fine wool sheep; LFT, Lanzhou fat-tailed sheep; II, insertion/insertion; ID, insertion/deletion; DD, deletion/deletion; HWE, Hardy-Weinberg equilibrium; He, heterozygosity; Ne, effective allele numbers; PIC, Polymorphism information content.

**Table 3:** Chi-square test of *HIAT1* P3-ins-9bp genotype distribution in the three sheep breeds.

**Breeds**	**LXBH**	**GSFW**	**LFT**
LXBH		7e-6^**^	8.6e-5^**^
GSFW	1.4e-5^**^		0.013^*^
LFT	4.24e-4^**^	0.038^*^	

Results below the diagonal line represent the Chi-square test of genotype frequency for each sheep breed; Results above the diagonal line represent the Chi-square test of allele frequency for each sheep breed.

* *P* < 0.05,

** *P* < 0.01.

### 3.3. Association between the P3-ins-9bp InDel and growth traits

The associations between the P3-ins-9bp InDel of the ovine *HIAT1* gene and growth performance were analyzed (Table 4). For LXBH sheep, t-tests results confirmed that the P3-ins-9bp InDel had no remarkable influence on growth traits for lambs (*P* > 0.05) (not shown). Yearling rams with the homozygous DD genotype were found to have lower chest depth, body weight, cannon circumference, and body height (*P* < 0.05). Yearling ewes with the homozygous DD genotype had significantly higher body weight, chest depth, chest circumference, height at the hip cross and abdominal circumference than those with the heterozygous ID genotype (*P* < 0.05). Additionally, adult ewes exhibited significant differences in height at the hip cross, body height, and cannon circumference (*P* < 0.01), with the heterozygous ID genotype being the dominant individual. For GSFW sheep, adult ewes with the heterozygous ID genotype showed superior performance compared to those with the homozygous DD genotype with regards to body weight, body length, chest width, and chest circumference.

**Table 4:** Association between the *HIAT1* P3-ins-9bp locus and sheep growth traits.

**Breeds**	**Types (n)**	**Traits**	**Observed genotypes (LSM** ±**SE)**		***P*-values**
LXBH	Yearling rams		ID (9)	DD (70)	
	(79)	Body weight (kg)	68.78 ± 7.00	54.47 ± 2.02	0.023
		Body height (cm)	70.67 ± 1.76	66.53 ± 0.68	0.041
		Chest depth (cm)	31.11 ± 1.15	27.63 ± 0.54	0.029
		Cannon circumference (cm)	10.22 ± 0.21	9.54 ± 0.11	0.036
	Yearling ewes		ID (16)	DD (82)	
	(98)	Body weight (kg)	39.99 ± 3.10	48.92 ± 1.52	0.018
		Height at the hip cross (cm)	64.38 ± 1.19	66.94 ± 0.51	0.044
		Chest depth (cm)	24.60 ± 0.83	27.47 ± 0.45	0.010
		Chest circumference (cm)	80.06 ± 2.20	86.87 ± 1.05	0.009
		Abdominal circumference (cm)	100.81 ± 3.07	108.52 ± 1.21	0.013
	Adult ewes		ID (15)	DD (58)	
	(73)	Body height (cm)	71.13 ± 1.31	68.05 ± 0.47	0.009
		Height at the hip cross (cm)	72.13 ± 1.18	69.16 ± 0.45	0.006
		Cannon circumference (cm)	10.20 ± 0.17	9.33 ± 0.12	0.001
GSFW	Adult ewes		ID (19)	DD (155)	
	(174)	Body weight (kg)	55.26 ± 2.00	49.81 ± 0.50	0.001
		Body length (cm)	82.68 ± 1.07	80.69 ± 0.31	0.037
		Chest width (cm)	30.05 ± 3.01	28.41 ± 0.18	0.005
		Chest circumference (cm)	99.63 ± 1.75	95.17 ± 0.49	0.004

## 4. Discussion

Recently, a study by Liu et al. (24) identified a quantitative trait locus (QTL) that affects milk traits in buffalo breed and includes *SLC35A3* (*Solute carrier family 35 member A3*) and *HIAT1* (24). The *SLC35A3* gene is located adjacent to the *HIAT1* gene and is involved in the transport of glucose, vitamins and other substances (25). A genome-wide association study (GWAS) on milk production traits determined that *SLC35A3* may also play a significant role in sheep milk traits (26). Moreover, a missense mutation in bovine *SLC35A3* has been found to lead to complex vertebral malformation (25). A recent study by Ye (15) found that *HIAT1* regulates milk fat synthesis in mammary epithelial cells through the PPAR signaling pathway, and that two SNPs within *HIAT1* are significantly associated with both milk fat and milk protein rates in buffalo (15). A more surprising finding is that both *HIAT1* and *SLC35A3* have the potential to affect both carcass and growth traits in chickens (27). In this study, we found that *HIAT1* has high amino acid sequence conservation, and that its tissue expression pattern is similar to that of humans. These results suggest that *HIAT1* might have a similar function amongst multiple types of mammals. Moreover, *HIAT1* mRNA expression was detected in perirenal fat, subcutaneous fat, and the livers of LFT sheep, suggesting that *HIAT1* may be related to fat deposition in sheep. These finding suggest *HIAT1* is a key candidate gene for regulating important economic traits in livestock.

Body morphology traits reflect the growth and development of different parts of the body and are also important economic and breeding indicators for both meat and meat-wool sheep breeds (28, 29). Existing studies have showed that the *HIAT1* gene influenced both growth and meat production traits in goats, and that a 15-bp insertion in *HIAT1* was associated with body morphology in Shaanbei white cashmere goats (19, 30). In this study, a 9-bp insertion mutation located in the 5'-upstream region of the ovine *HIAT1* gene (rs1089950828 -/GTCCAGTGG) was elucidated. It is well known that variations in the 5'-upstream region of genes has the potential to affect gene transcription and mRNA expression levels through changing transcription factor binding sites (TFBSs) and can also influence an individual's phenotype (31, 32). Thus, this research studied the association between the P3-ins-9bp InDel and sheep growth traits. Furthermore, the P3-ins-9bp InDel was strongly associated with body measurement traits in both LXBH and GSFW sheep based on association analysis. For yearling LXBH sheep, ewes with a heterozygous ID genotype demonstrated overall lower body size, while rams with a heterozygous ID genotype had better growth performance. These results indicate that the 9-bp insertion mutation may have a negative regulatory effect on both growth and development of LXBH yearling ewes as well as promote growth in yearling rams. Additionally, a heterozygous ID genotype in ewes was found to inhibit body size development in yearling sheep as well as promote growth in adult sheep. This may be due to the effect of the 9-bp InDel on the differential expression of *HIAT1* at different growth stages throughout the sheep lifecycle (33). Furthermore, the PIC value is an indicator of molecular marker quality in genetic studies. Our InDel locus of interest showed low polymorphism (*PIC* < 0.25) amongst all tested sheep according to the PIC value, indicating that there was sufficient space in which artificial selection can take place (34, 35).

**Figure 4:**
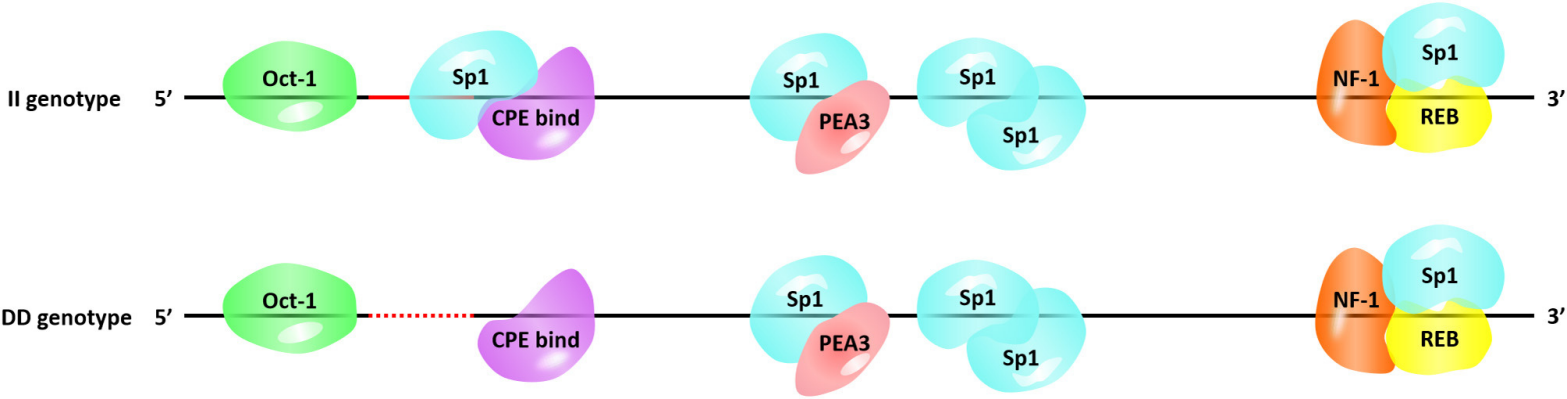
Transcription factor binding sites on the 9-bp InDel locus.

Next, we searched for the putative transcription factor binding sites that contained our InDel of interest. The P3-ins-9bp locus was found 624-bp upstream of the *HIAT1* transcriptional start site (TSS), which created an additional binding site for the transcription factor Sp1 (Specificity protein 1) falling upstream of the four existing Sp1 binding sites (Figure 4). SP1 is a transcription factor of the SP/KLF family. The regulation of SP1 with regards to target genes is achieved by binding of the DNA binding domain to GC-rich motifs in the promoter region of the target gene, thereby regulating cellular processes such as autophagy, apoptosis, proliferation, differentiation and angiogenesis (36, 37). Chen et al. (38) found that Sp1 binding sites in the core promoter region are essential for positive regulation of *FGF21* (*Fibroblast growth factor 21*) through gene transcription in both hepatic and adipose tissue (38). Furthermore, a recent study found that an SNP g.133A>C located within the SCD (*Stearoyl CoA desaturase*) promoter generated overall higher Sp1 binding affinity to the SCD promoter, which consequently affected gene expression (39). Moreover, a T > C mutation in the promoter region of *IGF1* (*Insulin-like growth factor 1*) was significantly associated with the litter size of Yunshang black goats. This mutation was found to create a new binding site for SP1, thereby promoting goat granulosa cell proliferation by regulating the expression of *IGF1* (40). Therefore, the interaction between Sp1 and *HIAT1* leads to changes in gene expression at both the mRNA and protein levels, which in turn has the potential to affect the growth traits of sheep. In future studies this interaction is worthy of further in-depth research and exploration. Furthermore, the molecular mechanism of action and associated allelic consequences should be further validated in future studies.

## 5. Conclusion

This study investigated both the mRNA expression profile and genetic variations of the ovine *HIAT1* gene. Our results demonstrated a high mRNA expression level of *HIAT1* in the testis of Lanzhou fat-tailed sheep. Moreover, a 9-bp insertion mutation (rs1089950828) in the 5'-upstream region of ovine *HIAT1* was detected. The frequency of the mutant allele “I” was found to be low. Furthermore, the 9-bp InDel locus was found to be closely associated with growth traits of LXBH and GSFW sheep, indicating that this InDel has the potential to be used as a genetic marker for assisted selection programs in domestic sheep.

## Data availability statement

The original contributions presented in the study are included in the article/supplementary material, further inquiries can be directed to the corresponding authors.

## Ethics statement

All animal experiments were conducted in accordance with the the China national standard of Guidelines on Welfare and Ethical Review for Laboratory Animals (GB/T 35892-2018). Our study was approved by Institutional Animal Care and Use Committee (IACUC) of Northwest A&F University.

## Author contributions

YL, XL, and HX came up with idea and revised the manuscript. YL wrote the manuscript and performed the experiments. CM, FJ, ZG, and HX collected the sheep samples and isolated the genomic DNA. YL and ZA analyzed the data. All authors approved the final version of the manuscript for submission, contributed to the article, and approved the submitted version.
